# American Medical Association

**Published:** 1850-03

**Authors:** 


*American Medical Association.*—Drs. C. B. Coventry, and Austin Flint have been elected by the Faculty of the Medical College of Buffalo, delegates to the next meeting of the American Medical Association.

